# Intra-arterial peptide receptor radionuclide therapy (IA-PRRT) in patients with SSTR-expressing neuroendocrine neoplasms: short- and long-term safety and efficacy for up to 13 years

**DOI:** 10.7150/thno.112012

**Published:** 2026-01-01

**Authors:** Jingjing Zhang, Birger Mensel, Richard P. Baum

**Affiliations:** 1 Department of Diagnostic Radiology, Yong Loo Lin School of Medicine, National University of Singapore, Singapore, 119074, Singapore.; 2 Theranostics Center of Excellence, Yong Loo Lin School of Medicine, National University of Singapore, 11 Biopolis Way, Helios, Singapore 138667, Singapore.; 3 Clinical Imaging Research Centre, Centre for Translational Medicine, National University of Singapore, Singapore 117599, Singapore.; 4 Nanomedicine Translational Research Program, Yong Loo Lin School of Medicine, National University of Singapore, Singapore 117597, Singapore.; 5 Department of Diagnostic & Interventional Radiology, Philipps-University Marburg, Germany; 6 Department of Diagnostic and Interventional Radiology and Neuroradiology, Central Hospital Bad Berka, Germany.; 7 Curanosticum Wiesbaden-Frankfurt, Department of Molecular Radiotherapy, ICPO Center of Excellence, DKD HELIOS Klinik, Wiesbaden, Germany.; 8 THERANOSTICS Center for Molecular Radiotherapy and Molecular Imaging, ENETS Center of Excellence, Zentralklinik Bad Berka, Bad Berka, 99437, Germany.

**Keywords:** Peptide receptor radionuclide therapy (PRRT), somatostatin receptor (SSTR), intra-arterial PRRT (IA-PRRT), ^177^Lu, ^90^Y, neuroendocrine neoplasms (NENs)

## Abstract

Intravenous peptide receptor radionuclide therapy (IV-PRRT) has established its role in the treatment algorithm of somatostatin receptor (SSTR)-expressing neuroendocrine neoplasms (NENs). This study aims to evaluate the safety and efficacy of intra-arterial PRRT (IA-PRRT) in patients with SSTR-expressing NENs.

**Methods:** The radiopharmaceutical was injected by a dedicated radionuclide infusion set *via* an intra-arterial catheter entering the femoral artery access site, with a microcatheter placed in the common hepatic artery or other selected artery *via* a standard access using the common femoral artery. Morphologic and molecular responses were evaluated in accordance with RECIST 1.1 and the EORTC criteria with ^68^Ga-SSTR PET/CT. Kaplan-Meier survival analysis was performed to calculate median progression-free survival (PFS) and overall survival (OS). Short- and long-term toxicities were documented in accordance with the CTCAE, version 5.0.

**Results:** 52 patients with SSTR-expressing NENs treated with intra-arterial PRRT with ^177^Lu- or ^90^Y-DOTATOC/DOTATATE from February 1999 to January 2019 were reviewed. The median follow-up time was 94.4 mo. Safety analysis demonstrated anemia (grade 1, n=4), leukocytopenia (grade 1, n=3; grade 2, n=1; grade 3, n=1), thrombocytopenia (grade 1, n=11) following IA-PRRT compared to baseline. No severe nephrotoxicity or liver dysfunction was observed after IA-PRRT. According to RECIST 1.1, the disease control rate at 3-6 mo after IA-PRRT was 89.4%, and the best objective response rate was 36.2%. For the entire cohort received IA-PRRT (n=52), the median PFS and OS were 29.9 and 68.9 months, respectively. In the subgroup of patients with neuroendocrine liver metastases receiving liver directed IA-PRRT, the median PFS and OS for patients with hepatic only tumor with or without lymph node metastases were significantly longer than those with extrahepatic-tumor (PFS, 35.9 mo *vs.* 21.6 mo, p=0.0128; OS, 80.1 mo *vs.* 50.5 mo, p=0.0470).

**Conclusions:** Intra-arterial PRRT is well-tolerated, safe and effective in patients with SSTR-expressing neuroendocrine neoplasms. The median OS and PFS appear promising, particularly in patients with hepatic tumor burden. No additional severe hematotoxicity, nephrotoxicity or hepatotoxicity was observed after IA-PRRT and during long-term follow-up. In particular, this procedure can be considered in patients with neuroendocrine liver metastases only or liver metastases mainly. Prospective studies are warranted to verify these results.

## Introduction

Neuroendocrine neoplasms (NENs) are a heterogeneous group of neoplasms arising from diffuse neuroendocrine system cells ranging from indolent well-differentiated neuroendocrine tumors (NETs) to aggressive poorly-differentiated neuroendocrine carcinomas (NECs) 1, 2. These tumors are characterized by high expression of somatostatin receptors (SSTRs), making them amenable to SSTR-directed imaging such as PET/CT using ^68^Ga-labeled somatostatin analogs and therapy with these analogs labeled with therapeutic radioisotopes such as β-emitters (*e.g.*, ^177^Lu or ^90^Y) or α-emitters (*e.g.*, ^213^Bi or ^225^Ac) for peptide receptor radionuclide therapy (PRRT) 3-10.

Over the past two decades, PRRT with β-emitters (^177^Lu or ^90^Y) labeled somatostatin analogs (DOTATATE or DOTATOC) has demonstrated remarkable success in the management of NETs 11-13. The significant benefit in terms of progression-free survival and response rates of PRRT over cold somatostatin analog therapy demonstrated by the randomized, controlled NETTER-1 trial led to the approval of ^177^Lu-DOTATATE (Lutathera) by both the European Medicines Agency and the U.S. Food and Drug Administration for the treatment of SSTR-positive gastroenteropancreatic neuroendocrine tumors (GEP-NETs) in adults 7. Quality of life is also significantly improved 14.

However, the overall tumor response rate comprising complete and partial remission after PRRT is still limited. A substantial portion of patients would remain stable disease after treatment. The objective response rate from NETTER-1 trial was 18% in NET patients after intravenous ^177^Lu-DOTATATE PRRT. Therefore, to maximize the full therapeutic potential of PRRT in NENs, more efforts are being investigated to further increase tumor dose delivery for highly effective tumor cell killing and more selective killing of targeted cancer cells while sparing surrounding healthy tissues.

The commonly used and currently established administration route is intravenous PRRT (IV-PRRT). As compared to IV-PRRT, selective intraarterial (IA) application of radiolabeled somatostatin analogs may provide intensifying therapy by delivering more concentrated doses to the tumor, particularly in patients with focal targeted primary tumor or liver metastases which is the most common site of GEP-NETs 15-17. A study from Heidelberg demonstrated increased tumor uptake of ^68^Ga-DOTATOC, in both the primary tumor of 1.44- to 7.8-fold and liver metastases of 3.75-fold higher uptakes, after selective IA administration in comparison with IV injection in neuroendocrine neoplasms 18. In addition to the increased tumor uptake, selective IA application of radiolabeled somatostatin analogs is also hypothesized to reduce the dose delivery to normal organs and thus further reduce the toxicity of the treatment 19-22. However, data on IA-PRRT for the treatment of NENs are very limited. Dosimetric analyses of IA versus standard IV administration indicated that IA administration of ^177^Lu-DOTATATE resulted in higher concentration and absorbed dose in hepatic metastases in patients of GEPNETs as compared to IV-PRRT, and thus seemed to be a powerful tool to improve the efficacy of PRRT 23. Limouris *et al.* 24 and Kratochwil *et al.* 25 reported promising therapeutic outcome with a high rate of tumor response in NET patients treated with arterial infusion of ^177^Lu-DOTA-TATE, and ^90^Y-/^177^Lu-DOTATOC, respectively. In the latter study, median time to progression was not reached within a mean follow-up period of 20 months 25.

The aim of this study was to assess the safety, covering both short-term toxicity and long-term toxicity, and efficacy, in terms of response rate and survival analysis, of IA-PRRT in patients with SSTR-expressing NENs.

## Materials and Methods

### Patients

All patients received ^177^Lu- or ^90^Y-DOTATOC/DOTATATE IA-PRRT under the compassionate use clause of the German Medicinal Products Act. This retrospective study was performed in accordance with German regulations (Federal Agency for Radiation Protection) concerning radiation safety and was approved by the local ethics committee (Bad Berka, Germany). All patients had undergone multiple lines of treatment, including surgery, long-acting somatostatin analogues, chemotherapy, previous IV PRRT, etc. IA-PRRT was done as part of a sequence of PRRT, *i.e.*, with systemic IV-PRRT during previous or following treatment courses. Decision to treat the patients by IA-PRRT was taken by internal or external tumor boards. All patients were either progressive before IA-PRRT, as determined by morphological imaging (CT or MRI) or by ^68^Ga-SSTR PET/CT, or were severely symptomatic due to extensive tumor mass or functional syndromes. All patients signed a detailed written informed consent form before undergoing the treatment, as well as consenting to the use of their anonymized clinical data for scientific purposes.

### IA-PRRT Treatment Regimen

The DOTA-conjugated somatostatin analogs DOTATOC and DOTATATE were labeled with ^68^Ga for SSTR PET imaging and either ^177^Lu or ^90^Y for IA-PRRT, in accordance with current good manufacturing practice (CGMP) regulations 26. More details are shown in the supplementary material.

**Figure 1:**
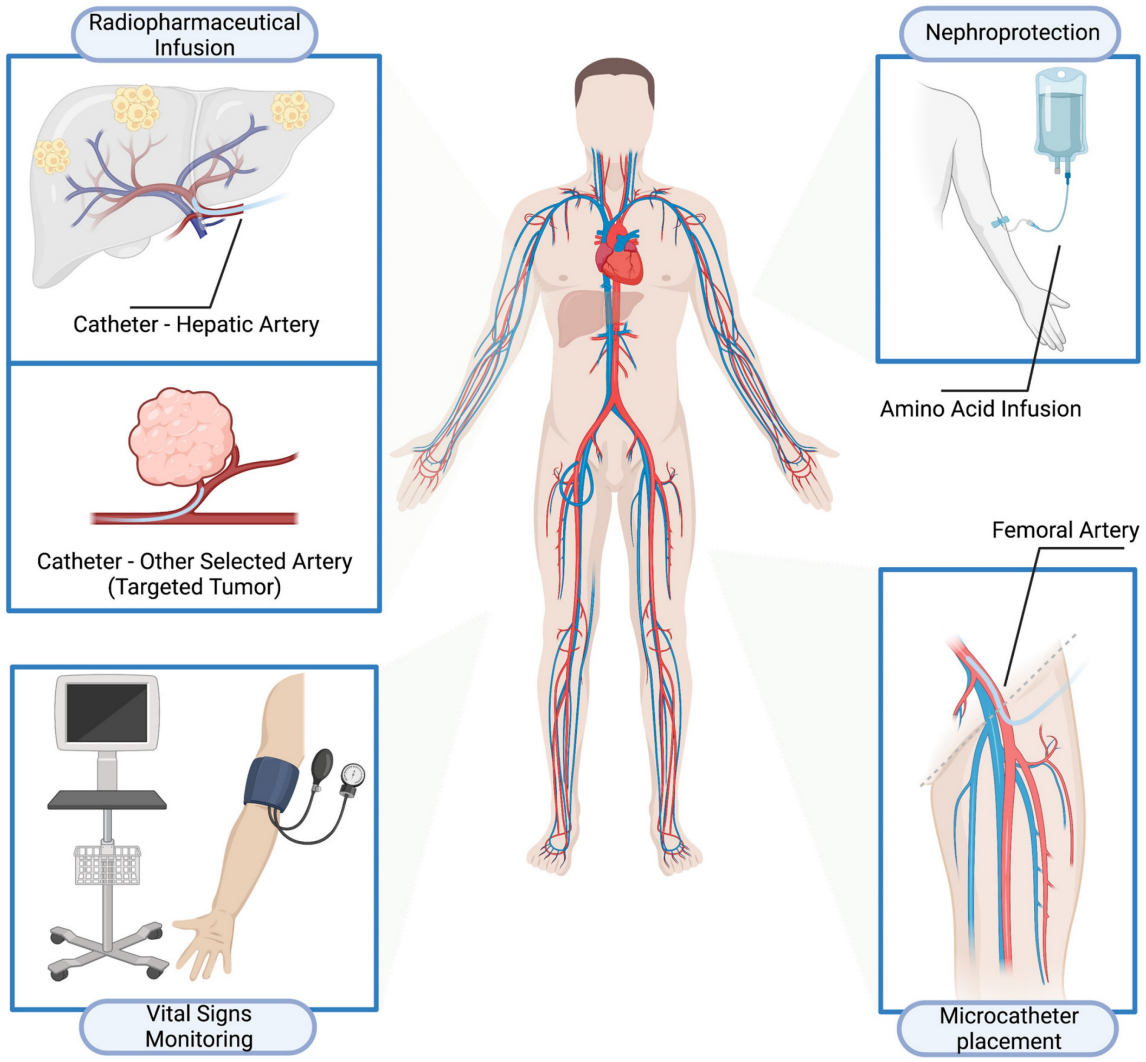
Treatment procedures of intra-arterial peptide receptor radionuclide therapy (IA-PRRT).

Pre-medication was pursued with intravenous glucocorticoid and antiemetic in adequate doses. The patient was transferred to the Angio suite. A microcatheter was placed in the common hepatic artery (for liver metastases) or other selected artery (targeted tumors at other sites, *i.e.*, primary tumors) *via* a standard access using the common femoral artery, by an interventional radiologist. The standard amino acid infusion for nephroprotection commenced intravenously *via* a peripheral venous cannula about 30 min before the intraarterial injection of the therapeutic dose of radionuclide and lasted for 4 h. Under direct medical supervision, the radiopharmaceutical was injected by a dedicated radionuclide infusion set over 60 min as slow infusion *via* the intra-arterial catheter entering the femoral artery access site. The patients were observed clinically, vital parameters (heart rate, blood pressure, temperature, *etc*.) were monitored and recorded at regular intervals before, during, and after the infusion (**Figure 1).** The intraarterial catheter was removed by a member of the interventional radiology team according to protocol, and usually about 6 h after insertion. A pressure bandage was placed on the intraarterial insertion site, which was monitored clinically, and removed as per interventional radiology protocol. The administered radioactivity was individually calculated on the basis of the Bad Berka Score; SUV on receptor PET/CT (referrals: OctreoScan K.S.), renal function (GFR and TER / creatinine & BUN), hematological status (blood counts), liver involvement, extrahepatic tumor burden, Ki-67 index/tumor grade, FDG status (glucose hypermetabolism of tumors), tumor dynamics (doubling time, new lesions), Karnofsky performance index, weight loss, time since first diagnosis and functional activity of tumor 27-31. Patients received IV and IA-PRRT at different stages in their disease; the time interval between cycles did not vary per se with the type of PRRT but was dependent on the Bad Berka score. For IA-PRRT, significant liver involvement, tumor progression, and heavy tumor burden of liver metastases or targeted tumors at other sites, *i.e.*, primary tumors, were the deciding factors.

### Toxicity Assessment

All patients were clinically monitored during therapy and for at least 2-4 days thereafter as inpatients for possible side effects. Vital parameters were recorded during therapy and a structured questionnaire documented any delayed complication. Laboratory analyses including hematologic status, renal function, and liver function were performed before and after IA-PRRT, and at each restaging. Details were prospectively documented in a structured database (comprising over 250 items per patient). Treatment-related adverse events were recorded in accordance with the National Cancer Institute Common Terminology Criteria for Adverse Events (CTCAE), version 5.0. Additional detailed parameters are shown in the supplementary material.

### Response Assessment

The treatment response was evaluated on CT or MR images according to Response Evaluation Criteria in Solid Tumors (RECIST 1.1) 32 and by PET imaging according to the criteria of the European Organization for Research and Treatment of Cancer (EORTC) 33. Imaging was performed before each IA-PRRT cycle and at restaging. Restaging was performed every 3-4 mo after each cycle of PRRT, and every 6 mo for stable disease or remission after initial follow-up, until disease progression was evident on imaging. The disease control rate was defined as complete remission (CR), partial remission (PR), and stable disease (SD). The best objective response rate was defined as patients achieving CR or PR at follow-up.

### Statistical Analysis

Kaplan-Meier survival analysis was performed to determine median progression-free survival (PFS) and median overall survival (OS), defined from the start of PRRT in general to a follow-up time of at least 3 mo after IA-PRRT. The median PFS and OS were compared to the previous cohort with comparable baseline characteristics receiving IV-PRRT at our center (**Supplemental Table 1**). The log-rank test was used to analyze the survival distribution of subgroups. Continuous variables were denoted as mean ± standard deviation. Differences between paired samples before and after treatment were determined by Student's t-test. For all variables that were proven with the Kolmogorov-Smirnov test to follow the skewed distribution, quantitative data were described in terms of median and range, and nonparametric sign tests were used to determine the significance of differences between parameters before and after treatment. All statistical tests were two-tailed, and a p value of less than 0.05 was considered statistically significant.

## Results

**Figure 2:**
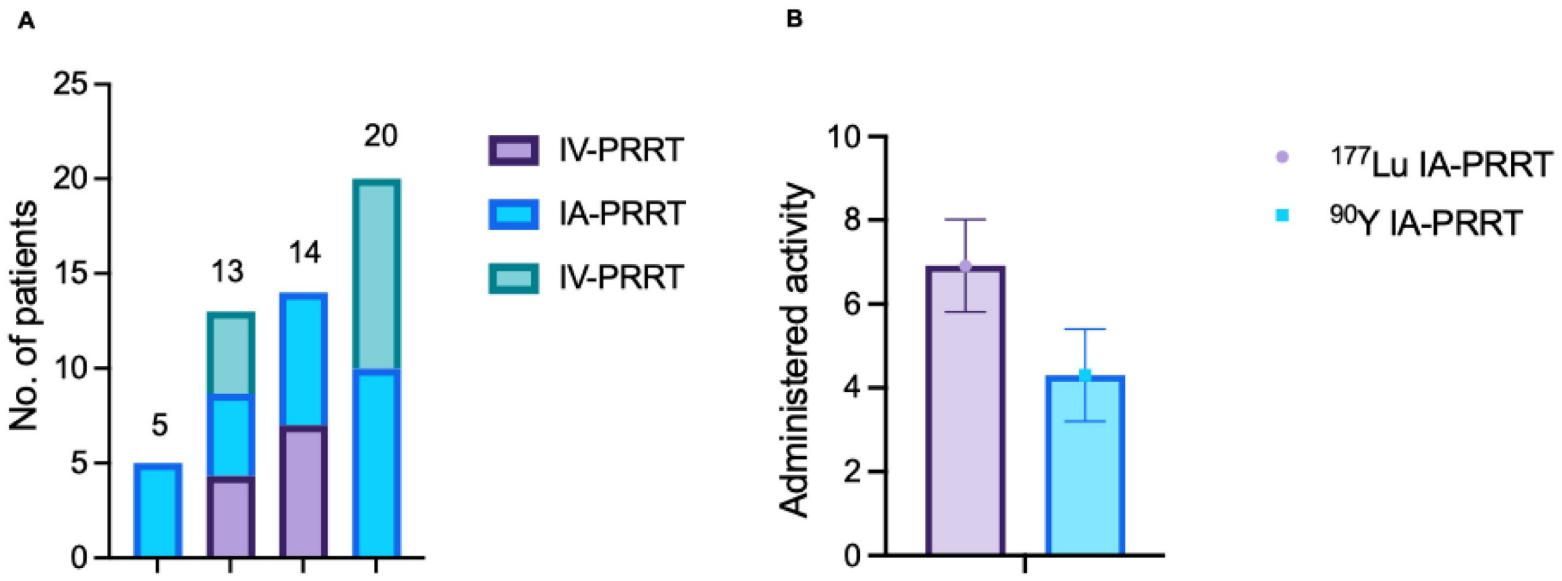
(A) Types of PRRT treatments. Five patients received IA-PRRT only; 14 patients received IV-PRRT followed by IA-PRRT; 13 patients received IV-PRRT, followed by IA-PRRT, and then IV-PRRT again; and 20 patients received IA-PRRT followed by IV-PRRT. (B) Median administered activity per cycle for IA-PRRT was 6.9 ± 1.1 GBq (range, 5.5 - 8.5 GBq) for ^177^Lu and 4.3 ± 1.1 GBq (range, 1.5 - 7.3 GBq) for ^90^Y.

Fifty-two patients (29 men and 23 women; age 19-80 y, mean age 52.4±14.4y) with metastatic SSTR-expressing NENs who received intra-arterial PRRT at Zentralklinik Bad Berka (Germany) from February 1999 to January 2019 were reviewed. The demographics of the patients at baseline are shown in **Table 1**. Twenty-seven patients (51.9%) were previously treated with systemic ^90^Y- or ^177^Lu- IV- PRRT during previous treatment courses. Among them, 13 patients also received IV-PRRT after at least one cycle of IA-PRRT. Five patients (9.6%) received IA-PRRT only, while 20 patients (38.5%) received IA-PRRT followed by IV-PRRT at different stages of their NEN disease (**Figure 2**). In total, 33 patients (63.5%) patients received IV-PRRT after at least one cycle of IA-PRRT.

The time interval between cycles did not vary per se with the type of PRRT. Treatment cycles and cumulative radioactivity are summarized in **Table 2**. The median administered activity for ^177^Lu IA-PRRT per cycle was 6.9±1.1 GBq (range, 5.5-8.5 GBq). The median administered activity for ^90^Y IA-PRRT per cycle was 4.3±1.1 GBq (range, 1.5-7.3 GBq). The maximum cumulative administered activities were 15.9 GBq and 22.6 GBq for ^177^Lu IA-PRRT and ^90^Y IA-PRRT, respectively.

### Safety

All patients tolerated the therapy without any serious acute adverse effects. No clinically significant adverse effects were noticed or reported by any patient during hospitalization for therapy or follow-up.

In the short-term following IA-PRRT, grade 3 leukocytopenia was observed in only 1 (1.9%) patient and subsequently improved to CTC grade 2 during follow-up. This patient, with a pancreatic NET, received 3 cycles of ^90^Y-IA-PRRT (intra-primary tumor, 3.6 GBq, 5 GBq and 2.5 GBq), followed by 1 additional cycle of ^177^Lu-IV-PRRT (5.6 GBq). Grade 3 leukocytopenia occurred after the first^ 90^Y-IA-PRRT and subsequently improved to CTC grade 2, which was maintained throughout follow-up.

During long-term follow-up with combined IA- and IV-PRRT, 1 patient (1.9%) developed grade 3 anemia. This patient had received 1 cycle of ^177^Lu-IV-PRRT (4.5 GBq), followed by 1 cycle of ^90^Y-IA-PRRT (4.5 GBq), and multiple cycle of ^177^Lu-IV-PRRT (5.4 GBq, 6.8 GBq, 5.6 GBq, 6.6 GBq). Grade 3 anemia occurred after the 6^th^ IV-PRRT cycle, approximately 16 months after the first IV-PRRT. Additionally, 1 patient (1.9%) developed grade 3 thrombocytopenia. This patient underwent 4 cycles of IV-PRRT (^177^Lu, 9 GBq; ^90^Y, 4.5 GBq; ^90^Y, 5 GBq; ^177^Lu, 5.7 GBq), 1 cycle of ^90^Y-IA-PRRT (7.3 GBq), and 2 further cycles of IV-PRRT (^177^Lu, 7.3 GBq; ^177^Lu, 5 GBq), CTC-3 thrombocytopenia occurred after the 6^th^ of IV-PRRT cycle, approximately 36 months after initiation of IV-PRRT.

**Figure 3:**
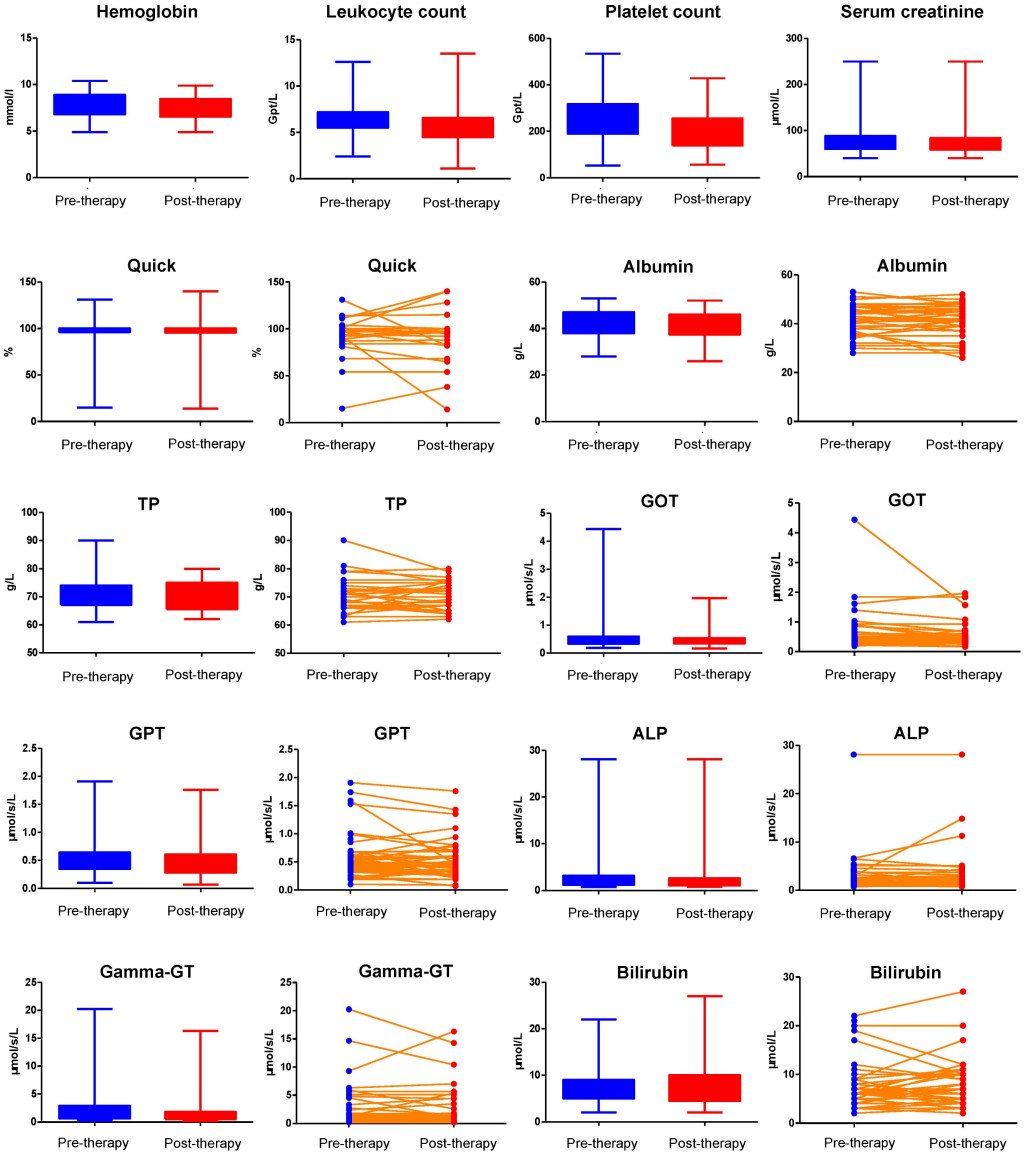
Comparison of hemoglobin, leukocyte count, platelet count, serum creatinine, Quick test (Prothrombin time/PT), albumin, total protein (TP), GOT, GPT, alkaline phosphatase (ALP), gamma-GT, and bilirubin before and after IA-PRRT.

No CTC grade 4 anemia, leukocytopenia or thrombocytopenia was observed. The hematological profile was shown in **Table 3**. Comparison of hemoglobin, leukocyte count, and platelet count before and after IA-PRRT was shown in **Figure 3**.

No CTCAE grade 3 or 4 nephrotoxicity was observed in any patient in the short-term following IA-PRRT. Furthermore, among patients presenting with a CTCAE grade 1 or 2 renal dysfunction before IA-PRRT, there was no additional post-therapeutic nephrotoxicity in any patient after IA-PRRT. During long-term follow-up with combined IA- and IV-PRRT, CTC grade 3 nephrotoxicity was documented in 1 of 52 patients (1.9%) with pancreatic NET. This patient had received 1 cycle of ^90^Y-IV-PRRT (4 GBq), 2 cycles of ^90^Y-IA-PRRT (intra-primary tumor, 3.5 GBq and 4.6 GBq), 1 additional cycle of^90^Y-IV-PRRT (5 GBq), and 1 further cycle of ^90^Y-IA-PRRT (3.5 GBq), resulting in a cumulative administered activity of 20.6 GBq between 2004 and 2005. On restaging follow up, CTC-3 nephrotoxicity was first detected in 2008, 4 years after the initiation of combined IA- and IV- PRRT. No CTC-4 nephrotoxicity was observed (**Table 4**).

There was no evidence of any severe hepatotoxicity during the short-term follow up after IA-PRRT. During long-term follow-up of patients treated with a combination of IA- and IV- PRRT, CTC-3 hepatotoxicity was observed in 2 of 52 patient (3.8%). One patient, who had received 1 cycle of ^90^Y-IV-PRRT followed by 1 cycle of ^90^Y-IA-PRRT and 2 cycles of ^177^Lu-IV-PRRT, developed grade 3 hepatotoxicity 18 months after initiation of combined IA+IV treatment, which subsequently improved to CTC-2. The second patient, who had initially received 1 cycle of ^90^Y-IA-PRRT without hepatotoxicity, later underwent 8 cycles of ^177^Lu-IV-PRRT and subsequently developed grade 3 hepatotoxicity 55 months after initiation of combined IA+IV treatment, likely attributable to significant disease progression of liver metastases. Importantly, no cases of CTC grade 4 hepatotoxicity were observed either in the short-term following IA-PRRT or in the long-term follow-up of patients receiving combined IA- and IV-PRRT **(Table 5)**. No evidence of significant synthetic liver dysfunction or any significant enzymatic hepatic dysfunction was observed. There was no significant change in albumin, total protein, Quick test (prothrombin time), AST, ALT, GGT, or bilirubin after IA-PRRT (p > 0.05) (**Figure 3).**

### Treatment Response

**Figure 4:**
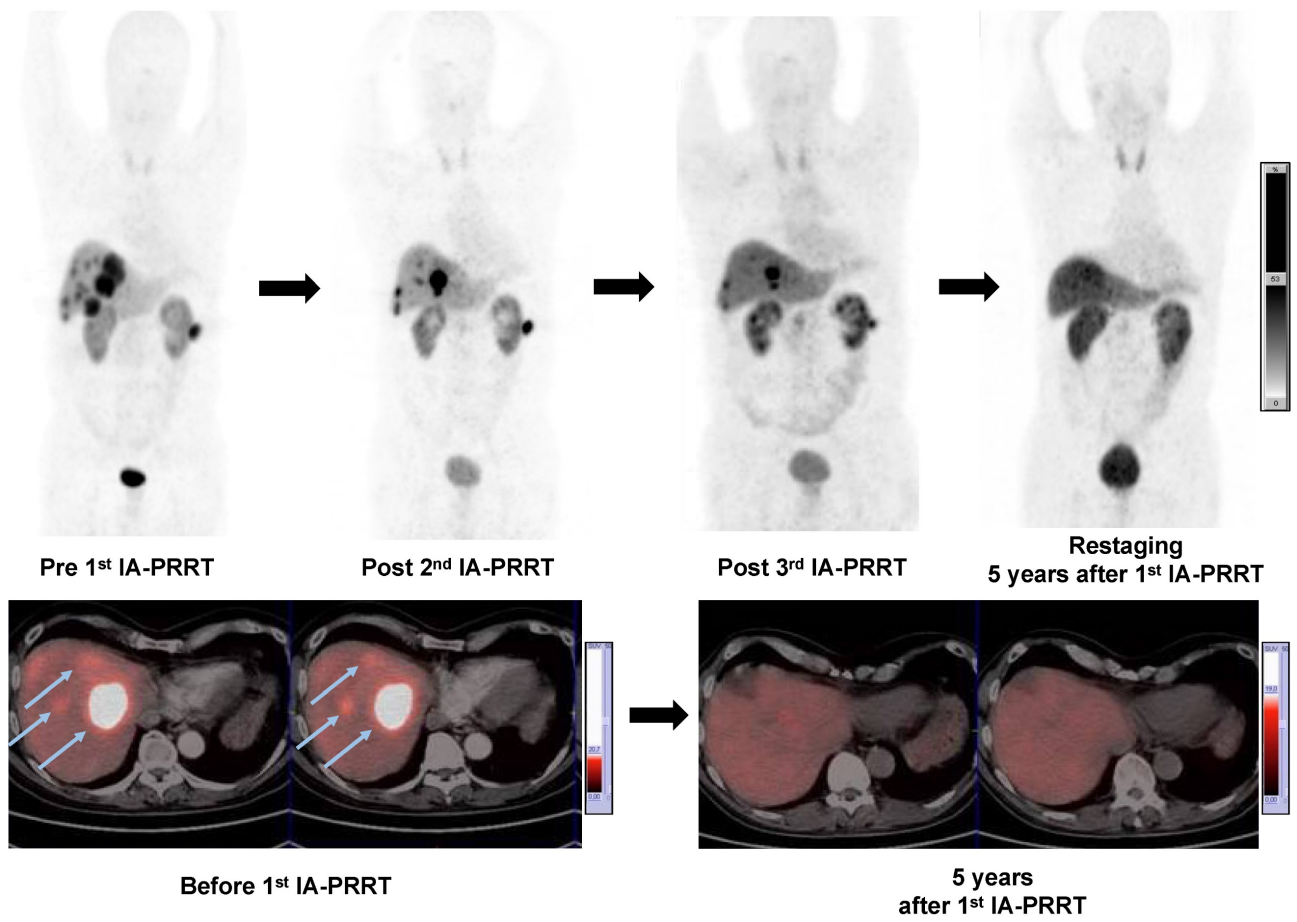
A 57-y-old man with well-differentiated, nonfunctioning metastatic pancreatic NEN. Maximum-intensity-projection (MIP) (top-left, Pre 1^st^ IA-PRRT) images from ^68^Ga-SSTR PET/CT showed SSTR expression in liver metastases (arrows) and lymph nodes metastases with SUVmax of 41.4. Patient was treated with 3 cycles of ^90^Y-DOTATATE IA-PRRT with cumulative administered radioactivity of 12.7 GBq. After the 2^nd^ IA-PRRT and 3^rd^ IA-PRRT, ^68^Ga-SSTR PET/CT showed significant regression of the liver and paracolic lymph node metastases (PR). MIP images from restaging ^68^Ga-SSTR PET/CT 5 years after 1^st^ IA-PRRT showed complete remission of the lesions. No adverse effects were observed during and after the treatment with long-term follow-up.

Of the 52 patients, a response evaluation at 3-6 mo after IA-PRRT was possible in 47 patients. According to RECIST 1.1, the disease control rate was 89.4%, including 1 (2.1%) patient with CR, 16 (34.0%) patients with PR and 25 (53.2%) patients with SD, whereas 5 patients (10.6%) had progressive disease (**Table 6**). The best objective response rate (ORR) was 36.2%. On the basis of the EORTC criteria, 1 (2.1%) patient had CR, 21 patients (44.7%) had PR and 20 patients had SD (42.6%). The disease control rate 3-6 months after IA-PRRT was 89.4%. The best objective ORR was 46.8%**. Figure 4** showed a representative example of tumor response after IA-PRRT.

In the long-term follow-up of patients who received both IA- and IV-PRRT, the best ORR and DCR were 48.0% and 90.0%, respectively, according to RECIST 1.1, and 66.0% and 90.0%, respectively, according to EORTC (**Table 7**). The median time to best observed response was 5.2 months for both EORTC (range, 1.8-86.6 months) and RECIST (range, 1.8-51.5 months). The best observed radiographic response among patients who did not receive IV-PRRT after IA-PRRT was an ORR of 64.7% and a DCR of 82.4% according to EORTC criteria, and an ORR of 41.2% and a DCR of 88.2% according to RECIST 1.1 (**Table 7**).

### Survival

**Figure 5:**
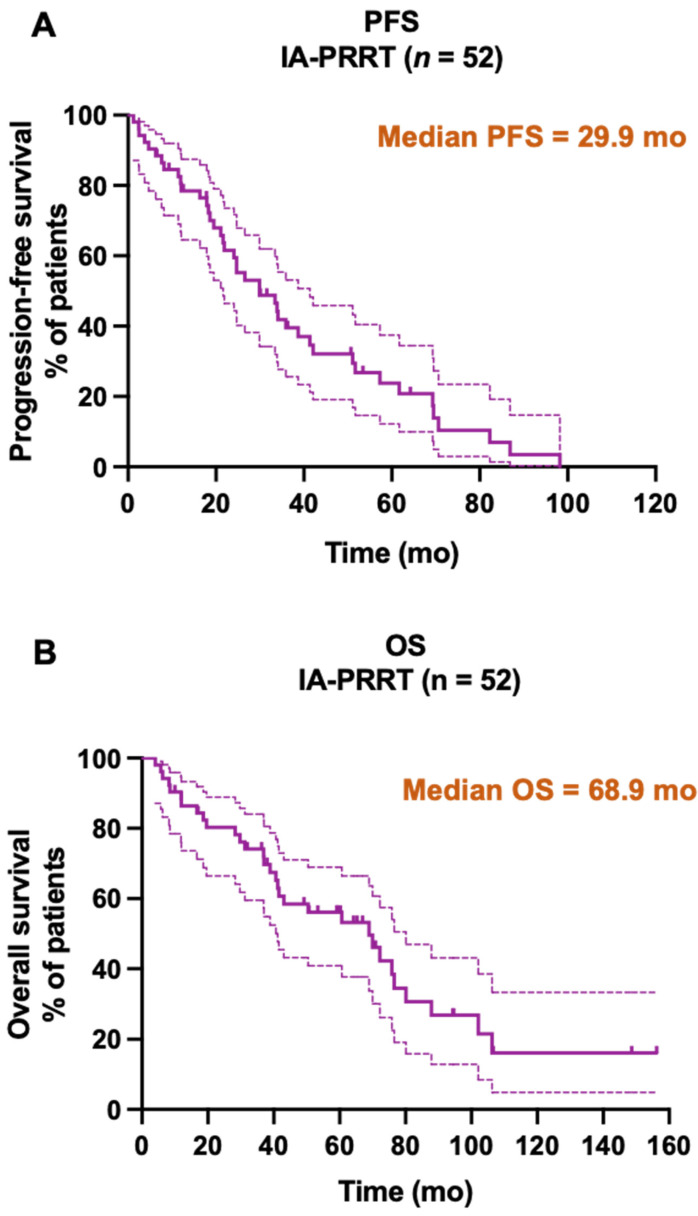
Kaplan-Meier curves for PFS (A, median PFS = 29.9 mo) and OS (B, median OS = 68.9 mo) from start of PRRT in general for all patients receiving IA-PRRT (not excluding those who also underwent additional IV-PRRT during the long-term follow-up period) in the present study (n = 52).

**Figure 6:**
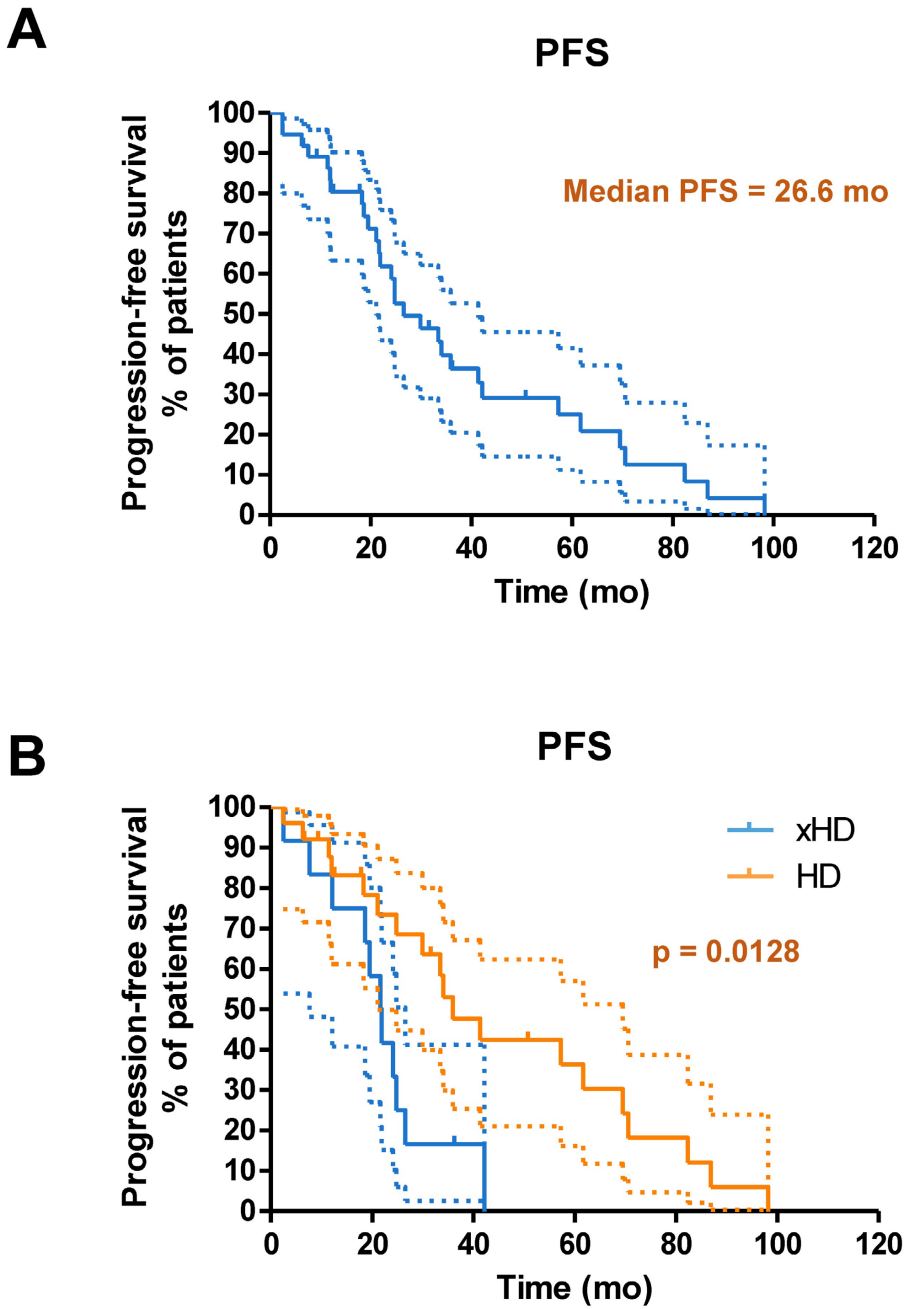
(A) Kaplan-Meier curves for PFS from start of PRRT in general for patients with neuroendocrine liver metastases receiving IA-PRRT (not excluding those who also underwent additional IV-PRRT during the long-term follow-up period) (n = 37). (B) Kaplan-Meier curves for PFS for patients with hepatic disease only (with or without lymph nodes metastases, HD, n = 25) and patients with extrahepatic tumor metastases (other organ involvement except lymph nodes, xHD, n = 12) (PFS, 35.9 mo *vs.* 21.6 mo, p = 0.0128).

**Figure 7:**
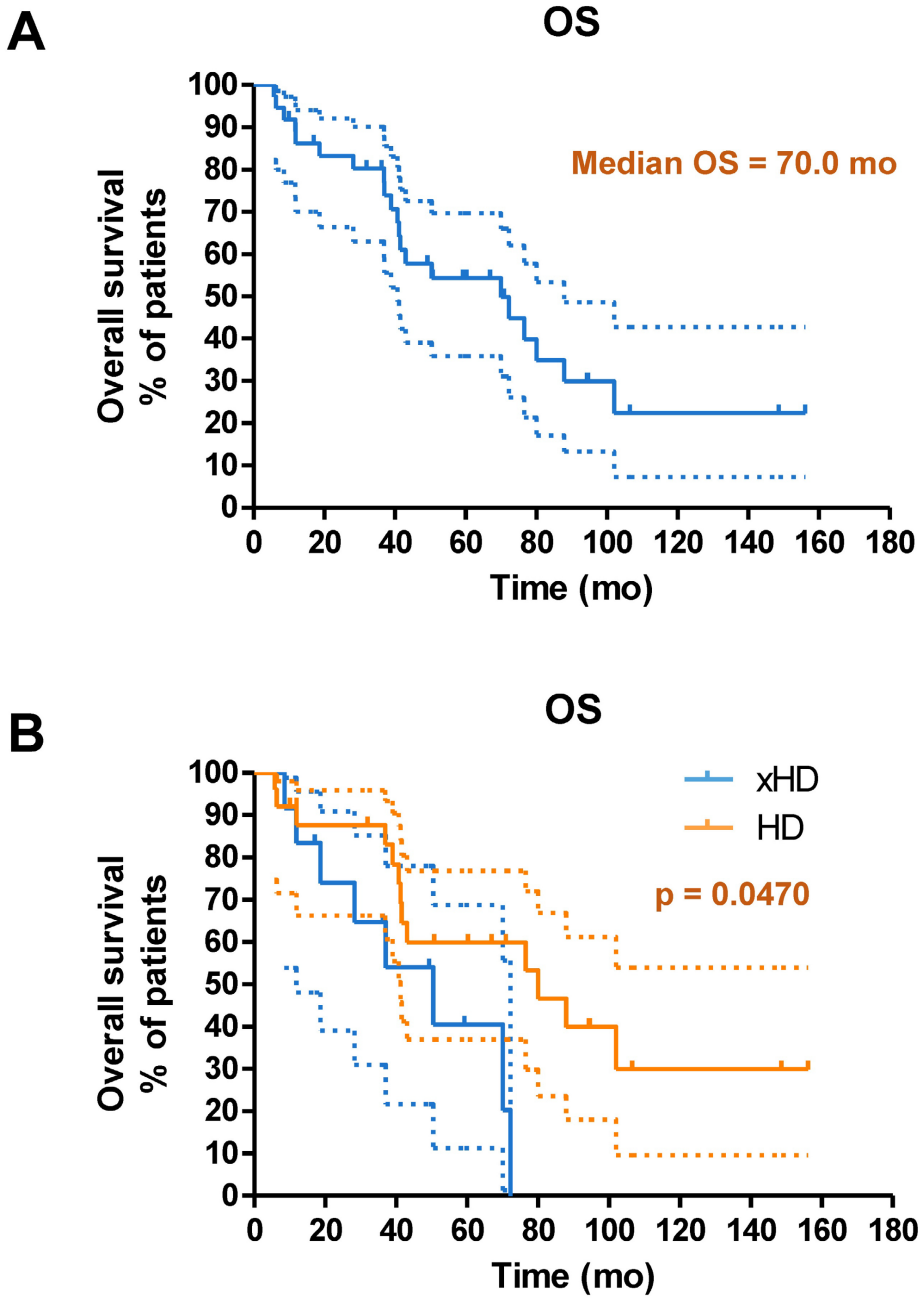
(A) Kaplan-Meier curves for OS from start of PRRT in general for patients with neuroendocrine liver metastases receiving IA-PRRT (not excluding those who also underwent additional IV-PRRT during the long-term follow-up period) (n = 37). (B) Kaplan-Meier curves for OS for patients with hepatic disease only (with or without lymph nodes metastases, HD, n = 25) and patients with extrahepatic tumor metastases (other organ involvement except lymph nodes, xHD, n = 12) (OS, 80.1 mo *vs.* 50.5 mo, p = 0.0470).

Until the study cutoff date in January 2019, 31 of 52 patients (59.6%) underwent IA-PRRT died. The median follow-up time was 94.4 mo (range, 4.0-156.2 mo). For the entire group of 52 patients receiving IA-PRRT (including those who also underwent additional intravenous PRRT during the extended long-term follow-up period), the median PFS and OS were 29.9 mo and 68.9 mo, calculated from the initiation of the first PRRT (**Figure 5**). For the 37 patients with neuroendocrine liver metastases (NELM) who underwent IA-PRRT and additional IV-PRRT treatments, the median PFS and OS were 26.6 mo and 70.0 mo, respectively, from the initiation of the first PRRT (**Figures 6 and 7**).

## Discussion

To our knowledge, this is the largest reported cohort of patients with SSTR-expressing NENs treated with IA-PRRT to date. Both short-term safety and responses after IA-PRRT, and long-term safety and survival accounted for by the additional treatments were evaluated. The follow-up (median, 94.4 mo) in this patient cohort is the longest among all published relevant studies. IA-PRRT resulted in excellent tumor response with a disease control rate of 89.4%.

The excellent treatment response to IA-PRRT was likely attributable to the combination of direct therapeutic effect of the radiopharmaceutical to the dominant tumors and systemic therapeutic effect to all the SSTR-expressing tumors. Kratochwil *et al.* reported a head-to-head intraindividual comparison of selective arterial versus venous ^68^Ga-DOTATOC PET/CT in 15 patients with GEP-NETs. They demonstrated several-fold higher uptake in the primary tumor as well as 3.75-fold higher uptake in liver metastases of NENs after selective IA administration in comparison with IV injection. Pool *et al.* also demonstrated increased radionuclide uptake of SSTR analog ^111^In-DTPAOC by using IA administration *via* the hepatic artery as compared to IV in NET liver metastases 34. Dosimetry studies further demonstrated high intratumoral concentration and prolonged absorbed tumor doses with IA administration 23, 35.

A more recent study by Lawhn-Heath *et al.* evaluated a single treatment using ^90^Y-DOTATOC and the comparison between IA and IV ^68^Ga-DOTATOC infusion in 5 patients, showing that IA ^68^Ga-DOTATOC failed to demonstrate increased uptake by hepatic metastases compared to IV 22. However, the time points differed between IA and IV infusion (63±7 min after IV injection *vs.* 90±20 min from the midpoint of the IA infusion), the result of which might not be suitable for a direct comparison; in addition, the IA ^68^Ga-DOTATOC could be influenced as it was administered concurrently with the therapeutic ^90^Y-DOTATOC. Lawhn-Heath *et al.* also reported in 10 patients that the single treatment using hepatic intraarterial administration of ^90^Y-DOTATOC did not induce tumor shrinkage, indicating that more treatment cycles might be required 22. In contrast, McStay *et al.* 20 and Kolasińska-Ćwikła *et al.* 21 reported hepatic intraarterial PRRT of ^90^Y-DOTA-lanreotide and ^90^Y-DOTATATE, being safe and effective for patients with progressive SSTR-positive liver metastases from NETs 20. Thakral *et al.* demonstrated that IA administration of ^177^Lu-DOTATATE resulted in higher concentration and absorbed dose in hepatic metastases of GEPNETs as compared to a single dose of PRRT administered through standard IV route. In a cohort with 12 patients, Limouris *et al.* reported that repeated, trans-hepatic high doses of ^177^Lu-DOTA-TATE resulted in a high tumor response with a PR in 75% of the treated patients in unresectable metastatic SSTR-positive liver lesions 24. Kratochwil *et al.* also reported the high rate of objective radiologic response in NET patients treated with arterial infusion of ^90^Y-/^177^Lu-DOTATOC compared favorably with systemic chemotherapy and intravenous radiopeptide therapy in a cohort of 15 patients with liver metastases arising from GEP-NETs *25*. More recently, a multicenter, randomized controlled trial in 26 NET patients is being conducted to investigate whether IA-PRRT with ^177^Lu-DOTATATE results in a higher activity concentration in liver metastases compared to IV administration 36. Our data are in accordance with the reported results, we observed a remarkably high response rate after IA-PRRT, likely due to the previously reported high first-pass effect, by delivering more concentrated doses of the agent to the dominant tumors, followed by a systemic therapeutic effect to other tumor locations as radioactivity is further distributed in the systemic circulation 18.

In the present study, both ^90^Y and ^177^Lu were used for IA-PRRT, with the concept of individualized precision oncology. Typically, we utilized ^90^Y for bulkier tumors due to the higher energy and ^177^Lu for smaller tumors. We also took into account the influence of the longer range of ^90^Y to normal organs such as bone marrow reserve, to maximize the patient benefits given the different physical properties of radioisotopes 12, 37. The administered radioactivity was individually calculated on the basis of the Bad Berka Score 12, 27, 28, 38, and the timing of IA-PRRT was related to disease spread as seen on PET/CT, especially regarding liver metastases. Although IA PRRT was not by itself an indication to give PRRT at a higher frequency, is not impossible that patients with more aggressive liver disease progression received an IA cycle earlier than they would have received an IV cycle had they had less aggressive disease.

The majority of patients in this cohort received IA-PRRT *via* hepatic artery. Accordingly, potential liver adverse effects could be expected. However, in this study, no severe (grade 3 or 4) hepatotoxicity was observed after 1-5 cycles of IA-PRRT. During long-term follow-up of patients treated with a combination of IA- and IV- PRRT, grade hepatotoxicity was observed in 2 of 52 patient. These events were transient, improving on subsequent follow-up, or were attributable to disease progression from liver metastases following IV-PRRT. Notably, no cases of grade 4 hepatotoxicity were observed either in the short-term after IA-PRRT or during long-term follow-up after combined IA- and IV-PRRT, underscoring the favorable safety profile of IA-PRRT.

The long-term outcomes of patients in the present cohort were very encouraging. For comparison, our previously reported cohort of 1048 patients who exclusively received intravenous PRRT at the same medical center, utilizing the identical EORTC criteria for PFS evaluation, had a median PFS and OS of 19 mo and 51 mo, respectively (**Supplemental Table 2**) 12. In contrast, the median OS and PFS in the present IA-PRRT cohort appeared to be improved compared with the prior standard IV-PRRT cohort. However, this study is limited by its nature as a single-center retrospective study, and the cohorts have not been case-control matched. Prospective studies are naturally needed in the future for further verification. In addition, we observed that as compared to patients with neuroendocrine liver metastases plus extrahepatic disease, patients with neuroendocrine liver metastases who underwent IA-PRRT and additional IV-PRRT treatments had a better outcome. These results might be partly attributable to the high first-pass effect of IA-PRRT treatment, by delivering more concentrated doses of the agent to the dominant tumors followed by the redistribution of radioactivity after the first pass effect in the systemic circulations as discussed above 18.

This study has a few limitations. One limitation of the present study is that it is a retrospective analysis (however, with prospective data sampling using a structured database). No strict pretest criteria for the selection of patients were applied, and the patient group was heterogeneous. Another limitation is the lack of availability of the exact Ki-67 index in 14 patients; these patients were referred from other centers and were characterized by histopathologically confirmed NENs and with tumor uptake on ^68^Ga-SSTR PET imaging. After IA-PRRT, per long-term follow-up, patients also received additional IV-PRRT given the remission of the dominant tumors after IA-PRRT or other consideration, and those patients were considered suitable for IV-PRRT at the given time. Therefore, the long-term outcome, in term of overall survival, was actually from the combination of IA-PRRT and IV-PRRT. There were variations in radioisotopes and SSTR affinities because different radiopharmaceuticals were used. Further prospective and controlled studies are certainly warranted.

## Conclusion

This study confirms that intra-arterial PRRT is well-tolerated, safe, and effective in patients with SSTR-expressing NENs. The median OS and PFS appear promising, particularly in patients with hepatic tumor burden. No additional severe hematotoxicity, nephrotoxicity, or hepatotoxicity as expected was observed after IA-PRRT and during long-term follow-up. Prospective studies are warranted to verify these results.

## Supplementary Material

Supplementary materials and methods, tables.

## Figures and Tables

**Table 1:** Demographic and baseline clinical characteristics of patients with NEN (*n* = 52)

Characteristics	Number (n)	Percentage (%)
Sex - no. (%)		
Male	29	55.8
Female	23	44.2
Age - yr	52.4±14.4	
Primary tumor site - no. (%)		
CUP	2	3.8
Pancreas	41	78.8
Midgut	6	11.5
Others	3	5.8
Functional *vs.* Nonfunctional - no. (%)		
Functional NEN	16	30.8
Nonfunctional NEN	36	69.2
Ki-67 index grading		
G1 (Ki-67 <3%)	10	19.2
G2 (Ki-67 =3%-20%)	26	50.0
G3 (Ki-67 >20%)	2	3.8
NA	14	26.9
Primary Tumor Resection		
Yes	22	42.3
No	30	57.7
Tumor metastases		
Liver	37	71.2
Lymph nodes	22	42.3
Lung	1	1.9
Peritoneum	2	3.8
Bone	8	15.4
Others	7	13.5
Microcatheter placement and administration routes for IA-PRRT		
Primary tumors (selected artery)	15	28.8
Pancreas	13	25.0
Ileum	2	3.8
Liver metastases (hepatic artery)	35	67.3
Both primary tumor and liver metastases (hepatic artery and other selected artery)	2	3.8
Previous treatments		
Surgery	44	84.6
Cold somatostatin analogues	24	46.2
Chemotherapy (excl. TACE)	22	42.3
TACE	8	15.4
Immunotherapy	2	3.8
Other radiotherapy (incl. SIRT)	3	5.8
Other (RFA, cryotherapy, other studies)	8	15.4

**Table 2:** Treatment cycles and cumulative administered radioactivity for IA-PRRT (*n* = 52)

Variables	n	%	Cumulative radioactivity (GBq)	
			Mean	SD
Number of IA-PRRT cycles	52	100		
1	38	73.1	4.8	1.3
2	6	11.5	9.4	4.1
3	5	9.6	13.6	4.1
4	0		0	/
5	1	1.9	22.6	/
TANDEM (^90^Y+^177^Lu)	2	3.8	11.8	/
Number of ^90^Y-IA PRRT cycles	42	80.8		
1	33	63.5	4.5	1.1
2	4	7.7	7.4	3.2
3	4	7.7	11.8	0.7
4	0		0	/
5	1	1.9	22.6	/
Number of ^177^Lu-IA PRRT cycles	6	11.5		
1	5	9.6	6.5	1.1
2	1	1.9	14.9	/
Number of ^90^Y+^177^Lu-IA PRRT cycles				
2	1	1.9	11.8	/
3	1	1.9	20.9	/
Number of TANDEM cycles	2	3.8		
1 cycle of TANDEM + 1 cycle of ^90^Y	1	1.9	15.4	/
1 cycle of TANDEM	1	1.9	8.1	/

**Table 3:** Hematotoxicity 3-6 months after the last cycle of IA-PRRT^§^ and long-term follow-up after IA-PRRT and IV-PRRT^*^ according to CTCAE v.5.0 (*n* = 52)

Numbers of patients with:									
	Anemia			Leukocytopenia			Thrombocytopenia		
Grade	Before IA-PRRT	After IA PRRT^§^	Long-term^*^	Before IA-PRRT	After IA PRRT^§^	Long-term^*^	Before IA-PRRT	After IA- PRRT^§^	Long-term^*^
CTC-1	28	32	31	5	8	6	2	13	11
CTC-2	5	7	10	1	2	3	1	1	0
CTC-3	0	0	1	0	1	0	0	0	1
CTC-4	0	0	0	0	0	0	0	0	0
CTC-5	NA	0	0	NA	0	0	NA	0	0

NA=not applicable before IA-PRRT (grade 5 represents death).

**Table 4:** Nephrotoxicity 3-6 months after the last cycle of IA-PRRT^§^ and long-term follow-up after IA-PRRT and IV-PRRT^*^ according to CTCAE v.5.0 (*n* = 52)

Numbers of patients with:			
	Nephrotoxicity		
Grade	Before IA-PRRT	After IA PRRT^§^	Long-term^*^
CTC-1	6	5	6
CTC-2	1	1	3
CTC-3	0	0	1
CTC-4	0	0	0
CTC-5	NA	0	0

NA=not applicable before IA-PRRT (grade 5 represents death).

**Table 5:** Hepatotoxicity 3-6 months after the last cycle of IA-PRRT^§^ and long-term follow-up after IA-PRRT and IV-PRRT^*^ according to CTCAE v.5.0 (*n* = 52)

Numbers of patients according to:															
	Albumin			Quick		AST		ALT		ALP		GGT		Bilirubin	
Grade	Before IA-PRRT	After IA PRRT^§^	Long-term^*^	After IA PRRT**^§^**	Long-term^*^	After IA PRRT^§^	Long-term^*^	After IA PRRT^§^	Long-term^*^	After IA PRRT^§^	Long-term^*^	After IA PRRT^§^	Long-term^*^	After IA PRRT^§^	Long-term^*^
CTC-1	4	4	6	0	0	1	2	0	0	0	0	0	1	0	0
CTC-2	1	3	3	1	0	0	1	0	2	1	5	1	3	0	2
CTC-3	0	0	0	0	0	0	1	0	1	0	1	0	2	0	0
CTC-4	0	0	0	0	0	0	0	0	0	0	0	0	0	0	0
CTC-5	NA	0	0	0	0	0	0	0	0	0	0	0	0	0	0

NA=not applicable before IA-PRRT (grade 5 represents death).

**Table 6:** Treatment response at 3-6 months after IA-PRRT (*n* = 47)

Response after IA-PRRT (n=47)	Total		including TANDEM*		^90^Y-IA PRRT	
	n	%	n	%	n	%
**EORTC - SSTR imaging response - no. (%)**	n=47		n=9		n=38	
Complete response	1	2.1	1	11.1	0	0
Partial response	21	44.7	6	66.7	15	39.5
Stable disease	20	42.6	2	22.2	18	47.4
Progressive disease	5	10.6	0	0	5	13.2
ORR	22	46.8	7	77.8	15	39.5
DCR	42	89.4	9	100	33	86.8
**RECIST - CT and/or MRI response - no. (%)**	n=47		n=9		n=38	
Complete response	1	2.1	1	11.1	0	0
Partial response	16	34.0	3	33.3	13	34.2
Stable disease	25	53.2	5	55.6	20	52.6
Progressive disease	5	10.6	0	0	5	13.2
ORR	17	36.2	4	44.4	13	34.2
DCR	42	89.4	9	100	33	86.8

* Numbers of patients underwent ^177^Lu-IA PRRT, ^90^Y+^177^Lu-IA PRRT or TANDEM PRRT

**Table 7:** Best tumor response after IA-PRRT and IV-PRRT in all patients (*n* = 50) and in patients who did not receive IV-PRRT after IA-PRRT (*n* = 17)

Response after IA-PRRT and IV-PRRT	Total	
	n	%
**EORTC - SSTR imaging response - no. (%)**	**n=50**	
Complete response	2	4.0
Partial response	31	62.0
Stable disease	12	24.0
Progressive disease	5	10.0
ORR	33	66.0
DCR	45	90.0
**RECIST - CT and/or MRI response - no. (%)**	n=50	
Complete response	2	4.0
Partial response	22	44.0
Stable disease	21	42.0
Progressive disease	5	10.0
ORR	24	48.0
DCR	45	90.0
**Response among patients who did not receive IV-PRRT after IA-PRRT**	**Total**	
	**n**	**%**
**EORTC - SSTR imaging response - no. (%)**	**n=17**	
Complete response	1	5.9
Partial response	10	58.8
Stable disease	3	17.6
Progressive disease	3	17.6
ORR	11	64.7
DCR	14	82.4
**RECIST - CT and/or MRI response - no. (%)**	**n=17**	
Complete response	1	5.9
Partial response	6	35.3
Stable disease	8	47.1
Progressive disease	2	11.8
ORR	7	41.2
DCR	15	88.2

## Abbreviations

NET: neuroendocrine tumor
NEN: Neuroendocrine neoplasm
NEC: neuroendocrine carcinoma
SSTR: somatostatin receptor
IA-PRRT: Intra-arterial peptide receptor radionuclide therapy
IV: intravenous
PET/CT: positron emission tomography combined with computed tomography
GEP-NETs: gastroenteropancreatic neuroendocrine tumors
NELM: neuroendocrine liver metastases
CUP: cancer of unknown primary
DOTA: 1,4,7,10-tetraazacyclododecane-N,N',N'',N'''-tetraacetic acid
LM4: p-Cl-Phe-cyclo[DCys-Pal-Daph(Cbm)-Lys-Thr-Cys]DTyr-NH2
SUV: standardized uptake value
CTCAE: Common Terminology Criteria for Adverse Events
RECIST: Response Evaluation Criteria in Solid Tumors
EORTC: European Organization for Research and Treatment of Cancer
CR: complete remission
PR: partial remission
SD: stable disease
PFS: progression-free survival
OS: overall survival
ORR: objective response rate
