# Supplementary material for: Intra-arterial peptide receptor radionuclide therapy (IA-PRRT) in patients with SSTR-expressing neuroendocrine neoplasms: short- and long-term safety and efficacy for up to 13 years
Source: Theranostics. 2026 Jan 1;16(4):1658–70. doi: 10.7150/thno.112012 (PMC12680495; doi:10.7150/thno.112012)
Supplement: Supplementary file 1 — Supplementary materials and methods, tables. [file thnov16p1658s1.pdf]

Intra-arterial peptide receptor radionuclide therapy (IA-PRRT) in patients with SSTR-expressing neuroendocrine neoplasms: short- and long-term safety and efficacy for up to 13 years

Jingjing Zhang[1,2,3,4*], Birger Mensel[5,6], Richard P. Baum[7,8*]

Supplementary Data

MATERIALS AND METHODS

IA-PRRT Treatment Regimen

The DOTA-conjugated somatostatin analogs DOTATOC and DOTATATE were labeled with ^{68}Ga for SSTR PET imaging and either ^{177}Lu or ^{90}Y for IA-PRRT, in accordance with good-manufacturing-practice regulations. ^{177}Lu and ^{90}Y were obtained from different manufacturers. Quality control parameters were monitored (radiochemical purity, radiochemical identity, pH value, ethanol content, endotoxin content, and proof of sterility). High-performance liquid chromatography was used for quality control. The radiochemical purity was always greater than 98%.

Post-therapy whole-body scintigraphy was performed with a SPIRIT DH-V dual-head gamma-camera (Mediso Medical Imaging Systems) using medium-energy general-purpose collimator, a 15% energy window with a peak at 208 keV, and a scan speed of 15 cm/min at 5 time points. SPECT/CT imaging was obtained approximately 24 hours after injection.

Toxicity Assessment

All patients were clinically monitored during therapy and for at least 2-4 days thereafter as inpatients for possible side effects. Vital parameters were recorded during therapy and a structured questionnaire documented any delayed complication. Laboratory analyses including hematologic status, renal function, and liver function were performed before and after IA-PRRT, and at each restaging. Details were prospectively documented in a structured database (comprising over 250 items per patient).

Kidney function was assessed by tubular extraction rate (TER) determined by ^{99m}Tc-mercaptoacetyltriglycine (MAG3) renal scintigraphy, glomerular filtration determined by ^{99m}Tc-DTPA clearance, and serum creatinine. Liver function was assessed using the following: Quick test (thromboplastin time/TPZ), albumin, total protein, AST, ALT, alkaline phosphatase, gamma-GT, bilirubin, other coagulation parameters. Treatment-related adverse events were recorded in accordance with the National Cancer Institute Common Terminology Criteria for Adverse Events (CTCAE), version 5.0, by assessing all available laboratory measurements performed regularly until death. In addition, adverse events were documented in an additional dataset by incoming patient reports during follow-up. Clinical data were from the patient file as well as during patient registration by trained physician assistants. Data analysis was performed on anonymized data sets. All patients irrespective of tumor stage or prognosis, received an appointment for follow-up. When patients did not appear, their family and/or physicians were contacted. Date of death was reported by family physicians. No patient was lost to follow-up.

Response Assessment

In addition, MRI in selected cases (allergy to iodinated contrast or poor detectability of liver metastases on CT scan) and routine sonography were performed for additional diagnostic evaluation. ^{68}Ga-SSTR PET/CT was performed until January 2014 with a Siemens Biograph Duo and since then with a Siemens Biograph mCT Flow 64. Contrast-enhanced CT (spiral CT using a Biograph mCT Flow 64) was acquired after intravenous administration of 60-100 mL of nonionic iodinated contrast agent. The disease control rate was defined as complete remission (CR), partial remission (PR), and stable disease (SD). The best objective response rate was defined as patients achieving CR or PR at follow-up.

Supplemental TABLE 1. Comparison of baseline characteristics between patients receiving IA-PRRT (n = 52) and IV-PRRT (n = 1048)

**Characteristics**	Group A (n=52)	Group B (n=1048)	P
Sex - no. (%)			0.908
Male	29 (55.8)	593 (56.6)	
Female	23 (44.2)	455 (43.3)	
Age - no. (%)			0.209
≤ 60 yr	28 (53.8)	655 (62.5)	
>60 yr	24 (46.2)	393 (37.5)	
Primary tumor site - no. (%)			0.001
CUP	2 (3.8)	151 (14.4)	
Pancreas	41 (78.8)	383 (36.7)	
Midgut	6 (11.5)	315 (30.0)	
Others	3 (5.8)	198 (18.9)	
Functional vs Nonfunctional - no. (%)			0.215
Functional NEN	16 (30.8)	244 (23.3)	
Nonfunctional NEN	36 (69.2)	804 (76.7)	
Ki-67 index grading			0.372
G1 (Ki-67 <3%)	10 (19.2)	247 (23.5)	
G2 (Ki-67 =3%-20%)	26 (50.0)	399 (38.1)	
G3 (Ki-67 >20%)	2 (3.8)	67 (6.4)	
NA	14 (26.9)	335 (32.0)	
Previous therapies			0.084
0	12 (23.1)	119 (11.4)	
1	14 (26.9)	303 (29.0)	
2-3	18 (34.6)	417 (39.7)	
>3	8 (15.4)	209 (19.9)	
Microcatheter placement and administration routes for IA-PRRT			NA
Primary tumors	15 (28.8)	NA	
Pancreas	13 (25.0)		
Íleum	2 (3.8)		
Liver metastases	35 (67.3)		
Both primary tumor and liver metastases	2 (3.8)		
